# Cyberbullying Involvement, Resilient Coping, and Loneliness of Adolescents During Covid-19 in Rural China

**DOI:** 10.3389/fpsyg.2021.664612

**Published:** 2021-06-16

**Authors:** Ziqiang Han, Ziyi Wang, Yuhuan Li

**Affiliations:** ^1^School of Political Science and Public Administration, Shandong University, Qingdao, China; ^2^School of Government, Central University of Finance and Economics, Beijing, China

**Keywords:** cyberbullying, loneliness, adolescent, Covid-19, China, resilience

## Abstract

Cyberbullying involvement can lead to internal health issues, especially mental health problems. Different coping strategies may reduce or enhance the strengths between cyberbullying experience and mental health problems. In this study, we examined the correlations between cyberbullying involvement and loneliness among a group of children and adolescents during the Covid-19 pandemic in China, focusing on investigating the protecting effect of the resilient coping strategy. The results demonstrated that 86.68% of the students were not involved in cyberbullying activities, 8.19% were victims only, 1.89% was perpetrators only, and 3.24% were both victims and perpetrators. Compared with the non-involved, the victims-only group had a significantly higher degree of reported loneliness and a lower score of resilient coping, while the differences of the other groups were not significant. Resilient coping strategy can significantly reduce loneliness and play a mediating role between cyberbullying victimization and loneliness, but such mitigating effect was relatively weak. Besides, peer relations were the primary protective factors, and age was the primary risk factor of loneliness among the controlled variables. This study can enrich current knowledge of cyberbullying involvement and the psychological health among children and adolescents, especially in the context of the pandemic.

## Introduction

The ongoing Covid-19 pandemic is still affecting people worldwide, and the countermeasures like social distancing and school closure can result in increased social isolation and loneliness in children and adolescents (Loades et al., 2020; Smith and Lim, 2020). Moreover, loneliness is associated with various types of mental health problems, such as anxiety and depression (Okruszek et al., 2020), substance use (Segrin et al., 2018), and even premature mortality (Goosby et al., 2013; Cacioppo and Cacioppo, 2018; Loades et al., 2020). Though social distancing or physical distancing may not necessarily lead to loneliness, the prevalence of loneliness had become much higher during the Covid-19 pandemic in 2020 when compared to data from two years ago (often lonely: 18.3% vs. 8.5%; sometimes lonely: 32.5% vs. 28.6%), and young adults are particularly at risk (Bu et al., 2020). Therefore, there is a solid need to re-investigate the loneliness of children and adolescents, especially in the context of lockdown due to the pandemic (Weeks and Asher, 2012; Cacioppo and Cacioppo, 2018; Loades et al., 2020).

Another observation during Covid-19 is the intensive use of the Internet and the increasing violence against children (Dong et al., 2020; Fore, 2020). The disruption of life and school closure put children and adolescents at greater risk of exposure to violence, such as domestic violence and cyberbullying (Babvey et al., 2020; Fore, 2020). Cyberbullying refers to the acts intended to harm others who cannot defend themselves in cyberspace or using information communication technologies (ICTs; Langos, 2012; Ansary, 2020), and it is increasing with the deep involvement of the young generation in cyberspace because the intensive use of the Internet may make cyberbullying more prevalent than before. A recent cross-national review indicates that the prevalence of cyberbullying is increasing worldwide, and China has a relatively higher prevalence (23.0%), compared with other countries such as Australia (5.0%), Sweden (5.2%), and Germany (6.3%). Another review conducted in 2021 also demonstrates that China ranked the fourth (44.5%) of the covered countries regarding the prevalence of cyberbullying victimization (Zhu et al., 2021). Internet accessibility and cultural differences can be the reasons for such differences, and the calculation methods of cyberbullying involvement may also contribute to such prevalence variations (Chen et al., 2004; Brochado et al., 2017; Heu et al., 2019; Zhu et al., 2021). For example, with the fast development of Internet infrastructure in China, 99.2% of the children and adolescents were found to access the Internet frequently in 2020, and 78% of them had started to use the Internet service under the age of 10 (Ji and Shen, 2020). Cyberbullying involvement can also lead to various mental health, social-psychological, and behavioral problems (Kwan et al., 2020), and loneliness can be one of such results (Segrin et al., 2012; Jiang et al., 2020). Considering the double impact of Covid-19 and cyberbullying on children and adolescents and the ongoing pandemic, there is a strong need to study the correlations between cyberbullying involvement and loneliness.

Coping strategies matter in dealing with violence exposure and mental health problems, especially during the Covid-19 pandemic (Cauberghe et al., 2020; Mariani et al., 2020; Yang, 2021). A recent survey from China showed that the problem-focused coping strategy was associated with less cyberbullying perpetration behavior but not the depression symptom, while the emotion-focused coping strategy was positively associated with both depression and cyberbullying perpetration behavior (Yang, 2021). Another study on coping strategies and mental health issues in Italy indicated that the emotion-focused coping strategies correlated with higher anxious and depressive symptoms (Mariani et al., 2020). Hence, the coping strategies should be considered when investigating the correlations between cyberbullying involvement and mental health problems, loneliness specifically in this paper.

Based on the discussion above, we assume that the lockdown during the Covid-19 pandemic increased the use of the Internet by children and adolescents, and thus, there may be an increase in cyberbullying involvement and feeling of loneliness. Moreover, coping strategies, especially the positive and resilient coping strategy (Fung, 2020), can reduce the associations between cyberbullying involvement and loneliness. Employing data consisting of 1,111 children and adolescents from one county in Shandong province from China, we hypothesize that:

*H1*: Cyberbullying involvement (both being a perpetrator and being a victim) is positively correlated with loneliness.

*H2*: Cyberbullying involvement (both being a perpetrator and being a victim) is negatively associated with the resilient coping score.

*H3*: Resilient coping score is negatively correlated with the degree of loneliness.

## Materials and Methods

### Participants and Sampling

We collected the data from one county in Shandong province, China. The county was purposively selected due to our connection with the local education district. We randomly selected one primary school, one middle school, and one high school from the list of the schools within the county with the help of the local education agency. Then, we reached out to one coordinator from each school, and all of them were the vice-principals of the schools. Considering the limited cognitive ability of young kids under grade three, we covered all the grade four, grade five, grade six, and all the middle school and high school students within the three selected schools.

The survey was distributed through an online survey platform.^1^ A link or QR code of the questionnaire was sent to all students through emails and social media apps such as WeChat, so the students can access the questionnaire and finish the survey independently. With help from the coordinators, we first established one temporary WeChat chatting group, including all the headmasters of the classes involved. The headmaster plays a unique and essential role in the Chinese education system. One headmaster is designated to one class within each school, from primary to middle and high schools. A headmaster plays dual roles in instructing a course and the management of all the students within the class. One headmaster usually has the contact information of the parents of all the students, and WeChat is one of the most commonly used communication tools in China. In most situations, there is at least one WeChat group including the parents of all the students and the headmaster for each class, so the WeChat group can be used to disseminate notices and information regarding all the school-related activities. Thus, the headmaster of each class distributed the link to the survey in their WeChat group, and the students can finish the survey using their phones or those of their parents.

The description of this study was distributed with the questionnaire to the parents and students as well. When the students opened the survey, the first page was the description of the study and the gratitude from the research group. On this page, the respondents were informed that all the participation process was voluntary and anonymous, and their parents should be aware of and agreed to this survey. Only the students who confirmed this by clicking the “Yes” option participated in the survey, and finally, 1,116 students participated in the survey, and 1,111 finished all the questions. Among the 1,111 participants, 54.91% of them were boys, 28.8% were primary school students, 43.74% were middle school students, and 27.45% were high school students.

### Measures

#### Cyberbullying Involvement

Based on the prior studies conducted by the authors (Han et al., 2017; Ba et al., 2019; Chai et al., 2020; Gong et al., 2020), the School Crime Supplement to the National Crime Victimization Survey developed by the National Center for Education Statistics of the United States (Lessne and Yanez, 2016) and the recent reviews of cyberbullying measurements (Berne et al., 2013; Chun et al., 2020), we inquired about the involvement of the adolescents in six types of cyberbullying behaviors: mocking, spreading lousy information or rumors, posting private information, threatening others, isolating others, and faking to be others in the cyberspace. For the subjects that were being cyberbullied, we asked the question, “Have your classmates or peers implemented these actions to you since January 2020?” The six statements were “Being mocked, called bad nicknames in cyberspace, including in the social media platforms like Weibo, WeChat, QQ, Tik Tok, or through SMS (short messages) or telephone calls”; “Somebody spread bad news or rumors about you in cyberspace”; “Posted your privacy information or photos or videos in cyberspace intentionally”; “Threatened you in cyberspace in chatting rooms or through social media, SMS”; “Isolated or Excluded you in cyberspace such as online games or chatting”; and “Hacked your online account or faked as you in cyberspace and did bad things.” The original answers to these questions were “never,” “rarely,” “sometimes,” and “frequently,” and we recoded the “never” as not being bullied (0), the “rarely,” “sometimes,” and “frequently” as being bullied in that specific manner (1) in our analysis. If a student was bullied by any of the six proposed actions, we defined the student to be cyberbullied. Otherwise, we defined them as not being cyberbullied. We did not differentiate the degrees of being cyberbullied in this analysis. As shown in Table 1, 11% of the students had been cyberbullied in 2020, 7% of them had been mocked, 5% had been victim to the spreading of rumors, 5% had been isolated, 4% had been faked, 3% had their private information posted, and 2% had been threatened in the cyberspace.

**Table 1:** Cyberbullying involvement and loneliness.

	Mean	*SD*	Min	Max	Cronbach
Bullied-Mock	0.07	0.26	0.00	1.00	0.8244
Bullied-Rumor	0.05	0.22	0.00	1.00	
Bullied-Privacy	0.03	0.17	0.00	1.00	
Bullied-Threat	0.02	0.14	0.00	1.00	
Bullied-Isolate	0.05	0.21	0.00	1.00	
Bullied-Fake	0.04	0.18	0.00	1.00	
Bullied	0.11	0.32	0.00	1.00	
Bully-Mock	0.02	0.15	0.00	1.00	0.9061
Bully-Rumor	0.02	0.15	0.00	1.00	
Bully-Privacy	0.02	0.15	0.00	1.00	
Bully-Threat	0.02	0.13	0.00	1.00	
Bully-Isolate	0.03	0.16	0.00	1.00	
Bully-Fake	0.02	0.13	0.00	1.00	
Bully	0.05	0.22	0.00	1.00	
Relation with classmates	4.66	0.66	1.00	5.00	
Relation with parents	4.70	0.69	1.00	5.00	
Relation with teachers	4.64	0.69	1.00	5.00	
Loneliness score	4.02	2.54	0.00	12.00	
I experience a general sense of emptiness	0.49	0.64	0.00	2.00	0.5971
I miss having people around	1.07	0.81	0.00	2.00	
I often feel rejected	0.46	0.65	0.00	2.00	
There are plenty of people I can rely on when I have problems (reversed)	0.70	0.75	0.00	2.00	
There are many people I can trust completely (reversed)	0.72	0.74	0.00	2.00	
There are enough people I feel close to (reversed)	0.59	0.72	0.00	2.00	
Resilient coping score	15.68	4.14	4.00	20.00	
I look for creative ways to alter difficult situations	3.79	1.21	1.00	5.00	0.9011
Regardless of what happens to me, I believe I can control my reaction to it	3.83	1.20	1.00	5.00	
I believe that I can grow in positive ways by dealing with difficult situations	4.03	1.16	1.00	5.00	
I actively look for ways to replace the losses I encounter in life	4.02	1.14	1.00	5.00	

Similarly, we inquired about the cyberbullying behaviors of the students to others. The question “Have you implemented the following behaviors to your classmates or other peers since January 2020?” was used, and the same statements and measurement strategies provided to the being-cyberbullied group were used. As shown in Table 1, the self-reported cyberbullying perpetration behaviors were about half of the self-reported being-cyberbullied subjects. About 5% of the students reported that they had cyberbullied others. About 3% of the respondents indicated that they had isolated others in cyberspace, while the prevalence of mocking others, spreading rumors about others, releasing private information about others, threatening others, and faking to be others online was 2%.

We conducted the confirmatory factor analysis (CFA) and principal component analysis (PCA) to test the convergence feature of the six cyberbullying victimization variables and the six cyberbullying perpetration variables. For the cyberbullying victimization scale, the cumulative explained variance of factor one from PCA was 0.5556, while the CFA results were optimal (overall *R*^2^ = 0.888, RMSEA = 0.165, CFI = 0.913, TLI = 0.856, SRMR = 0.057, and CD = 0.888). For the cyberbullying perpetration scale, the cumulative explained variance of factor one from PCA was 0.6871, while the CFA results were good as well (overall *R*^2^ = 0.938, RMSEA = 0.098, CFI = 0.982, TLI = 0.969, SRMR = 0.021, and CD = 0.938). The Cronbach’s alpha test results for cyberbullying victimization and cyberbullying perpetration were 0.8244 and 0.9061, respectively, demonstrating excellent internal reliability.

As shown in Table 2, 86.68% of the respondents had not been involved in cyberbullying, 3.24% of them were both victims and perpetrators of cyberbullying, and 8.19% of the students were victims only, while the last 1.89% was self-reported perpetrators only.

**Table 2:** Characteristics of the respondents.

	Frequency	Percent (%)
**Cyberbully involvement**		
Not Involved	963	86.68
Victim only	91	8.19
Perpetrator only	21	1.89
Victims and Perpetrator	36	3.24
**Gender**		
Female	501	45.09
Male	610	54.91
**School**		
Primary	320	28.8
Middle	486	43.74
High	305	27.45
**Single-Child**		
No	818	73.63
Yes	293	26.37
**Economic ranking**		
Poor	63	5.67
Median	872	78.49
Rich	176	15.84
**Academic**		
Low	35	3.15
Low-median	138	12.42
Median	296	26.64
Upper-median	410	36.9
Upper	232	20.88
**Living with**		
Parents	969	87.22
Only father	25	2.25
Only mother	32	2.88
Grandparents	69	6.21
Others	16	1.44
**Father job**		
Farmers-agricultural	423	38.07
Farmer-outside	327	29.43
Full-time	325	29.25
Jobless	36	3.24
**Mother job**		
Farmers-agricultural	569	51.22
Farmer-outside	165	14.85
Full-time	308	27.72
Jobless	69	6.21
**Father education**		
Middle or lower	609	54.82
High	406	36.54
College+	96	8.64
**Mother education**		
Middle or lower	693	62.38
High	312	28.08
College+	106	9.54
**Marriage**		
Others	100	9
First marriage	1,011	91
Total	1,111	100

#### Loneliness

The Chinese version of the 6-item De Jong Gierveld Loneliness Scale (Gierveld and Tilburg, 2006; Leung et al., 2008) was employed to measure loneliness in this survey. It has six items, and three of them captured emotional loneliness, while the other three measured social loneliness. The question used was “how the statement below can represent your current emotions?” and the six statements were “I experience a general sense of emptiness,” “I miss having people around,” “I often feel rejected,” “There are plenty of people I can rely on when I have problems,” “There are many people I can trust completely,” and “There are enough people I feel close to.” The answers to each of the statements were No (0), More or Less (1), and Yes (2). The social loneliness scales were recoded to represent the degree of loneliness in an increasing way, that is, a higher value of the scale means a higher degree of loneliness. The aggregation results of agreements to all the six statements were used as the score of loneliness, ranging from 0 to 12, while the sum of the first three was the score of emotional loneliness, ranging from 0 to 6, and the sum of the last three was the score of social loneliness. The overall loneliness score was 4.02 on average, with a standard deviation of 2.54, while the mean value for the emotion loneliness was 2.02 and for the social loneliness was 2.01. For individual statements, the ranking of average values of loneliness was missing people around (1.07), trust (0.72), dependent (0.70), intimate (0.59), empty (0.49), and rejected (0.46).

#### Resilient Coping

The brief resilient coping scale developed by Sinclair and Wallston was used in this study (Sinclair and Wallston, 2004). It has four items inquiring the perception of a respondent regarding their coping strategy after difficulties, and a validation of the Chinese version has demonstrated that it has good reliability and validity in the Chinese context (Fung, 2020). The question is, “how do you agree with the following statement?” and the answers are measured by 5-point Likert scale, ranging from 1 to 5 and representing an increasing degree of agreement. As shown in Table 1, the four statements were about “looking for creative ways to coping difficult situations,” “controlling reactions,” “growing positive ways of coping,” and “looking for ways to replace the losses.” The average ratings for the four statements were 3.79, 3.83, 4.03, and 4.02, respectively. The aggregation of the ratings of the four statements was used as the resilient coping indicator in the analysis, and thus, the resilient coping indicator had a mean value of 15.68, with a SD of 4.14, a minimum value of 4.0, and a maximum value of 20.

#### Social Relations

The self-reported social relations of the respondents with classmates, parents, and teachers were also included. The question was “overall, how do you evaluate your relationship with your classmates/parents/teachers?” and the answers were measured by 5-point Likert scale, representing the meaning from “badly” to “very good.” The average assessment of the relationships with classmates, parents, and teachers were 4.66, 4.70, and 4.64, and the SDs was 0.66, 0.69, and 0.69, respectively.

#### Control Variables

The essential socioeconomic and demographic variables like gender (male = 1), whether being a single-child at home (yes = 1), the marital status of the parents (first marriage = 1), main job of the parents (agriculture-related, migrant workers in cities, having full-time jobs, and jobless) and education status (middle school or lower, high school, college, and above), perceived socioeconomic ranking of family within the region (low, median, and rich), perceived academic performance (5-point Likert scale), the primary guardians living with (parents, father only, mother only, grandparents, and others), and school types (primary school, middle school, and high school) were included as the control variables. As shown in Table 2, 54.91% of the respondents were boys, 28.8% were primary school students, 43.74% were middle school students, and 27.45% were high school students. A total of 26.37% of the respondents were the only child within their family, 5.67% of them perceived their family to be poor, 78.49% reported middle level, and 15.84% thought they were rich. Most of the respondents (87.22%) were living with both parents, 2.25% were living with the father, 2.88% were living with their mother, 6.21% were living with the grandparents, and 1.44% was living with other relatives or friends. The perceived academic performance ranking in the five scales from low to high was 3.15, 12.42, 26.64, 36.9, and 20.88%.

### Data Analysis Strategy

We first reported the descriptive analysis of all the variables, including the detailed information of the cyberbullying involvements, resilient coping, and loneliness items. Since loneliness and resilient coping were treated as continuous scores, we used ordinary least squares (OLS) regression for analysis. The mediation effect of the resilient coping between cyberbullying involvement and loneliness was analyzed by the most widely used four-step method (MacKinnon et al., 2007). All the data analyses were implemented by the statistical software Stata 16.

## Results

We classified the involvement of the respondents in cyberbullying into four groups: the non-involved, the victim only, the perpetrator only, and both victims and perpetrators. We primarily examined the correlations between cyberbullying involvement and self-reported loneliness. Meanwhile, the mediating role of resilient coping between cyberbullying involvements was examined, and the results were reported in Table 3.

**Table 3:** Cyberbullying involvement, resilient coping, and loneliness (*N*=1,111).

	Bully→Lonely	Bully→Resilient	B+M→Lonely	Resilient→Lonely
**Cyberbullying involvement (not involved as reference)**				
Victim only	0.58^*^ (0.24)	−1.02^*^ (0.40)	0.50^*^ (0.24)	
Perpetrator only	−0.58 (0.47)	1.07 (0.80)	−0.49 (0.47)	
Victim and perpetrator	0.63 (0.38)	−2.04^**^ (0.64)	0.46 (0.38)	
Resilient			−0.14^***^ (0.02)	−0.15^***^ (0.02)
Relation classmate	−1.33^***^ (0.14)	0.44 (0.24)	−1.30^***^ (0.14)	−1.34^***^ (0.14)
Relation parents	−0.22 (0.13)	1.02^***^ (0.22)	−0.14 (0.13)	−0.13 (0.13)
Relation teachers	−0.17 (0.15)	0.70^**^ (0.26)	−0.12 (0.15)	−0.12 (0.15)
Male	−0.22 (0.14)	−0.51^*^ (0.23)	−0.26 (0.14)	−0.24 (0.14)
**School type (primary school as reference)**				
Middle school	0.42^**^ (0.16)	0.15 (0.27)	0.43^**^ (0.16)	0.47^**^ (0.16)
High school	2.59^***^ (0.27)	1.84^***^ (0.46)	2.74^***^ (0.27)	2.73^***^ (0.27)
Being a single child	0.40^*^ (0.19)	−0.52 (0.32)	0.36 (0.19)	0.35 (0.19)
Socioeconomic	0.11 (0.17)	−0.56 (0.29)	0.07 (0.17)	0.04 (0.17)
Academic	−0.12 (0.07)	0.35^**^ (0.12)	−0.10 (0.07)	−0.09 (0.07)
**Living with (parents as reference)**				
Father only	−0.02 (0.47)	−1.71^*^ (0.78)	−0.15 (0.46)	−0.20 (0.46)
Mother only	0.54 (0.40)	−1.38^*^ (0.68)	0.43 (0.40)	0.37 (0.40)
Grandparents	−0.00 (0.31)	−0.56 (0.53)	−0.05 (0.31)	−0.05 (0.31)
Others	0.26 (0.56)	−0.59 (0.95)	0.21 (0.56)	0.18 (0.56)
**Father’s job (agricultural as reference)**				
Migrant workers	0.03 (0.17)	0.42 (0.29)	0.06 (0.17)	0.05 (0.17)
Full-time jobs	−0.23 (0.27)	0.28 (0.46)	−0.21 (0.27)	−0.20 (0.27)
Jobless	0.44 (0.42)	0.43 (0.71)	0.47 (0.42)	0.48 (0.42)
**Mother’s job (agricultural as reference)**				
Migrant workers	0.21 (0.21)	0.06 (0.36)	0.22 (0.21)	0.22 (0.21)
Full-time jobs	−0.07 (0.28)	0.26 (0.47)	−0.05 (0.28)	−0.05 (0.28)
Jobless	−0.40 (0.32)	0.80 (0.53)	−0.33 (0.31)	−0.32 (0.31)
**Father education (middle or below as reference)**				
High school	0.28 (0.18)	−0.52 (0.30)	0.24 (0.18)	0.23 (0.18)
College or above	0.11 (0.33)	−1.24^*^ (0.56)	0.01 (0.33)	0.02 (0.33)
**Mother’s education (middle or below as reference)**				
High school	0.16 (0.20)	0.21 (0.34)	0.17 (0.20)	0.19 (0.20)
College or above	−0.68^*^ (0.33)	−0.30 (0.56)	−0.71^*^ (0.33)	−0.67^*^ (0.33)
Not first marriage	−0.24 (0.27)	2.10^***^ (0.46)	−0.07 (0.27)	−0.08 (0.27)
Adjusted *R*^2^	0.302	0.249	0.313	0.311

Standard errors in parentheses.

* *p* < 0.05;

** *p* < 0.01;

*** *p* < 0.001.

Overall, the results demonstrated that the victim-only group had a much higher degree of loneliness than the non-involved group when all the variables were included. The pure perpetrators did not exhibit significant differences, and the positive effect of the dual victims and perpetrators on loneliness was dismissed when the social relationships were included. The victimization in cyberbullying was associated with a lower degree of resilient coping, as shown in the Bully Resilient model. When the resilient coping variable was included in the model (B+M Lonely), the victim-only group had a 0.50 higher degree of loneliness compared with the non-involved group, as opposed to a 0.58 effect when the resilient indicator was not included (Bully Lonely).

The resilient coping strategy predicted a lower degree of loneliness, and being a cyberbullying victim or being a victim-perpetrator had a significantly lower degree of the resilient coping score. The resilient coping strategy did reduce the coefficients of cyberbullying involvement on loneliness. However, such protection effect was minimal. Moreover, social relationships, especially the relationship with peers, exhibited a significant protective role between cyberbullying involvement and loneliness.

Besides, age was the primary risk factor for loneliness. Compared with the students in primary schools, students in middle school and high school had higher degrees of reported loneliness, particularly high school students. The education of the mother played a protective effect. The loneliness did not show significant differences among the groups that reported different socioeconomic rankings, jobs of parents, education statuses of the father, marital statuses, and gender differences. Meanwhile, being a single child within a family and the status of living with both parents or single parent or grandparents did not affect the reported loneliness as well, overall.

## Discussion

The intensive Internet use during the Covid-19 pandemic can increase loneliness and violent behaviors toward children and adolescents, such as domestic abuse and cyberbullying, as well as other psychosocial health problems and even suicides (Goosby et al., 2013; Loades et al., 2020; Okruszek et al., 2020). This study examined the correlations between cyberbullying involvement and the feeling of loneliness using a sample of pre-college adolescents from rural China, and the role of the coping strategy between cyberbullying involvement and loneliness was also examined.

Cyberbullying prevalence did increase during the Covid-19 outbreak in 2020. About 11% of the respondents reported that they were cyberbullied, and 5% said they had cyberbullied others in 2020. Among the six types of cyberbullying behaviors, “mocking others in cyberspace” was the one with the highest prevalence rate (7%). Such prevalence rates are higher than previous reports of cyberbullying in China; a very recent survey conducted in 2019 from Jiangsu province showed that the self-reported cyberbullying victimization prevalence was 7.49%, while the perpetration rate was 2.05% (Zhang et al., 2020). Another survey conducted between 2009 and 2010 from Xi’an city revealed that the self-reported cyberbullying victimization in the last year was 6.3% (Zhu et al., 2019), while another large study conducted during a similar period also reported 5.51% of cyberbullying victimization (Chen et al., 2018).

The cyberbullying victimization experience is correlated with a higher degree of loneliness, but not the cyberbullying perpetration, so research hypothesis 1 is partially supported. Being a pure victim had a 0.50 higher degree of reported loneliness when all the potential confounding variables were included, and the pure perpetrator and the victim-perpetrator did not demonstrate such significant difference. There are very limited studies that have examined the correlations between cyberbullying experience and loneliness. In general, the quality and quantity of social networks and social support is the widely recognized determinant of loneliness (de Jong Gierveld, 1998). Some specific groups, such as the young adults, the elderly, women, people living alone, people with personal constraints related to social skills, people with low income, and residents in the urban environment, are at risk of loneliness (de Jong Gierveld, 1998; Beutel et al., 2017; Bu et al., 2020). Some recent studies have indicated that the experience of being victimized is associated with loneliness among the youth (Cole et al., 2021), and moreover, the victimization experience in childhood can lead to loneliness during childhood and even early adulthood (Matthews et al., 2020). Regarding cyberbullying in specific, only one study from Spain has reported positive correlations between cybervictimization, loneliness, and poor school adjustment (Cañas et al., 2020). For the controlled variables, similar to a previous review (Chai et al., 2019), peer relationship was the protective factor, while the older students tended to have a higher degree of loneliness in the analysis presented in this study, but the protective roles of other social support and relationships were not significant. Besides, we found that if the mother had an education attainment level of college or above, the adolescent would report a lower degree of loneliness.

Cyberbullying involvement is also partially associated with the resilient coping score. The pure victims and the subjects in the dual victim and perpetrator group adopted a much lower degree of resilient coping strategies, but the effect of the pure perpetrator was not significant. Prior studies have indicated that the emotional coping strategies are correlated with a higher degree of mental health problems, while the problem-focused coping strategy is not (Mariani et al., 2020; Yang, 2021), but we did not differentiate between the different coping strategies in our analysis. Moreover, we found the social relationships with peers, parents, and teachers, especially the relationship with peers, played more significant roles in reducing the impact of cyberbullying involvement and the feeling of loneliness, as a recent review indicated (Chai et al., 2019).

We have admitted that there are at least three limitations of this study. First, the inevitable limitation of a cross-sectional study cannot produce real causal relations. Second, we only employed a survey from one county in Shandong province, and thus, it limited the representativeness of the findings. For example, Chinese children in rural and urban contexts may have different experiences of loneliness (Chen et al., 2014). Third, we only included the resilient coping scale as the coping strategy measurement, and this measure neglected other negative coping styles. Considering the increasing integration of cyberspace and physical space in our daily life, more studies regarding violence in cyberspace are needed in the future.

In conclusion, we examined the correlations between cyberbullying involvement, resilient coping, and loneliness of a group of adolescents from rural China in the context of the Covid-19 outbreak in 2020. The results demonstrated that the cyberbullying victimization experience was correlated with a lower resilient coping strategy and a higher degree of loneliness, while the perpetration experience alone did not predict the two measures, and the ones with the dual role of victim and perpetrator had the lowest resilience coping score and the highest loneliness score. Moreover, a resilient coping strategy can reduce the correlations between cyberbullying involvement and loneliness, but such mitigation effect was limited.

## Data Availability Statement

The raw data supporting the conclusions of this article will be made available by the authors, without undue reservation.

## Ethics Statement

The studies involving human participants were reviewed and approved by the Ethics Committee of the School of Political Science and Public Administration, Shandong University, China. Written informed consent from the participants’ legal guardian/next of kin was not required to participate in this study in accordance with the national legislation and the institutional requirements.

## Author Contributions

YL contributed to the idea of this paper and data collection. ZW contributed to the data analysis and literature review. ZH contributed to the research design and manuscript preparation. All authors contributed to the article and approved the submitted version.

### Conflict of Interest

The authors declare that the research was conducted in the absence of any commercial or financial relationships that could be construed as a potential conflict of interest.

## Footnotes

^1^www.credamo.com
